# Live Cell Monitoring and Enrichment of Stem Cell‐Derived β Cells Using Intracellular Zinc Content as a Population Marker

**DOI:** 10.1002/cpsc.99

**Published:** 2019-10-28

**Authors:** Jeffrey C. Davis, Aharon Helman, José Rivera‐Feliciano, Christine M. Langston, Elise N. Engquist, Douglas A. Melton

**Affiliations:** ^1^ Department of Stem Cell and Regenerative Biology Harvard University Cambridge Massachusetts; ^2^ Harvard Stem Cell Institute Harvard University Cambridge Massachusetts; ^3^ Howard Hughes Medical Institute Chevy Chase Maryland

**Keywords:** intracellular zinc content, SC‐β cell enrichment, stem cell‐derived β (SC‐β) cell

## Abstract

Our laboratory and others have developed protocols to generate glucose‐responsive stem cell–derived β cells in vitro. The cells resulting from these protocols could supplement or replace the use of human cadaveric islets for cell‐based therapy for diabetes. The combination of an unlimited supply of pluripotent stem cell–derived β cells and gene‐editing approaches will facilitate numerous in vitro studies not possible with cadaveric islets. Here, we describe a protocol for fluorescent labeling and isolation of stem cell–derived β cells. This purification of SC‐β cells is based on intracellular zinc content and is a simple method to complement other approaches for generating and assaying these cells. © 2019 The Authors.

**Basic Protocol**: Fluorescent labeling and isolation of stem cell‐derived β cells

Protocols to generate SC‐β cells have been successfully performed in a number of laboratories (Nostro et al., 2015; Pagliuca et al., 2014; Rezania et al., 2014; Russ et al., 2015). These cells share a glucose responsiveness profile and similarity in gene expression to human cadaveric islets (Pagliuca et al., 2014; Veres et al., 2019). These protocols yield mixed populations of α, β, δ, and other cells types of the islet, as well as undifferentiated progenitors or smaller populations of undesired cells.

Insulin comprises approximately 10% of the total protein content within the β cell (Weir & Bonner‐Weir, 2013). This abundance of insulin requires efficient packing and storage in secretory vesicles, which is achieved in SC‐β cells (Pagliuca et al., 2014). Organization of insulin into dense core granules is facilitated by crystallization, seeded by two zinc ions at the center of each hexamer within the secretory vesicle (Dodson & Steiner, 1998). Thus, β cells have high levels of intracellular zinc and high expression of zinc transporters. Appropriate expression of zinc transporters and packaging of insulin have previously been confirmed in the SC‐β population (Pagliuca et al., 2014), and high intracellular zinc has been used to image and isolate the islet β cell population (Burdette, Frederickson, Bu, & Lippard, 2003; Latif, Noel, & Alejandro, 1988; Lukowiak et al., 2001; Meeusen, Tomasiewicz, Nowakowski, & Petering, 2011). Here, we describe the use of live‐cell zinc dyes for isolation and monitoring of SC‐β cells. Recent reports have also suggested that enrichment of the SC‐β population for extended culture may improve insulin secretory profiles of SC‐β cells using genetic reporters (Nair et al., 2019; Veres et al., 2019). Here, we describe a method to enrich SC‐β cells without the need for gene editing, allowing studies of SC‐β biology across multiple genetic backgrounds and human islets.

The dye *N*‐(6‐methoxy‐8‐quinolyl)‐*p*‐toluenesulfonamide (TSQ) offers a simple, broadly applicable method to analyze the SC‐β population across multiple backgrounds, as the TSQ^+^ population labels a large fraction (>80%) of the insulin^+^ population. This was measured using a differentiated genetic knock‐in reporter line, INS^mCherry^, for insulin expression, as analyzed by flow cytometry (Fig. 1A,B). Furthermore, reaggregation after sorting allows for homogenous clusters of similar size and intracellular zinc content to human islets (Fig. 1C). These cells can be cultured in 96‐well format, allowing large‐scale experiments with multiple conditions and a defined number of cells in each cluster.

**Figure 1:**
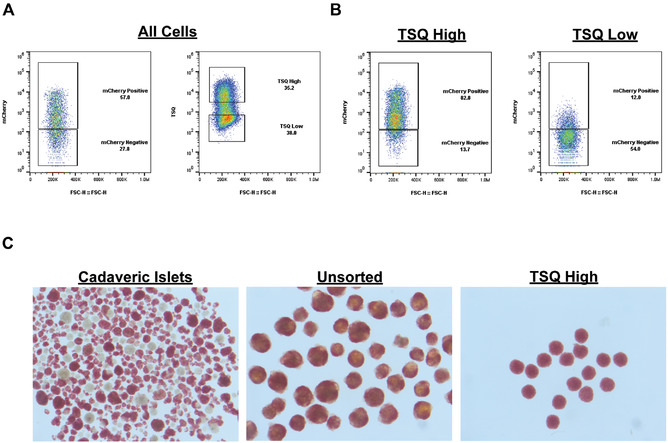
Analysis of SC‐β cells before and after enrichment and reaggregation using TSQ‐based FACS. (**A**) Flow cytometry analysis of 1016 INSmCherry knock‐in reporter line without sorting for mCherry expression (left) and TSQ live cell staining (right). (**B**) Analysis of mCherry expression in TSQ High (left) and TSQ Low (right) subpopulations. (**C**) Dithiazone staining of human cadaveric islets (left), unsorted SC‐β cells (middle), and reaggregated, TSQ‐enriched SC‐β cells (right) 72 hr post‐sort. Light microscopy images were taken at 2.5× on a dissecting light microscope.

## Fluorescent labeling and isolation of stem cell–derived β cells

The following protocol is useful to fluorescently label stem cell–derived β cells, allowing for rapid isolation of differentiated insulin‐producing cells without the need for gene‐edited reporter lines, and facilitates SC‐β cell‐specific analyses across many genetic backgrounds. This approach may enable studies with more broadly applicable conclusions by using genetically diverse pluripotent stem cell lines.

## Materials

Differentiated stem cell–derived β cells using the protocols reported by our laboratory (Pagliuca et al., 2014; Veres et al., 2019).
SC‐β cell culture medium (S3; see recipe)
10 mM Rho kinase inhibitor stock
Phosphate‐buffered saline (PBS; ThermoFisher, cat. 10010002)
Accutase cell dissociation reagent
25 mg/ml TSQ dye (Enzo Life Sciences, cat. no. ENZ‐52153) in DMSO; store protected from light (Meeusen et al., 2011)
1 mg/ml propidium iodide live/dead indicator (Sigma‐Aldrich, cat. no. P4864)
Sorting buffer (see recipe)

Centrifuge
40‐µm pore‐size sterile filter
Fluorescence‐activated cell sorting (FACS) instrument capable of UV/violet‐range detection
Multichannel pipettor and sterile trough for medium
12‐well aspirating manifold for changing medium (Drummond Scientific, cat. no. 3‐000‐096)
96‐well V‐bottom plates for culture of enriched, reaggregated SC‐β cells after isolation

1 Begin with stem cell–derived β cells differentiated by previously described protocols. Maintain cells at 1 × 10^6^ cells per ml of S3 culture medium.
2 Wash SC‐β cells in PBS and aspirate medium. Replace PBS with room‐temperature Accutase. Use 1 ml of Accutase per 5 × 10^6^ cells. Allow clusters to incubate at 37°C for 10 min.
3 Using a P‐1000 (1‐ml) pipet tip, thoroughly triturate the cluster mixture in Accutase until no visible cell clusters are left.
4 Quench reaction using a 20‐fold excess of medium followed by centrifugation for 3 min at 230 × *g*, room temperature, to pellet dissociated SC‐β clusters. Resuspend cells in PBS containing a 1:1000 dilution of the 10 mM stock of Rho kinase inhibitor.
5 Pipet to break up clumps of dissociated cells and pass the dissociated cell suspension through a 40‐µm pore‐size filter to remove larger clumps of undissociated and/or dead cells.
6 Dilute 25 mg/ml TSQ dye DMSO stock solution 1:2000 into the SC‐β cell suspension (final concentration being 12.5 µg/ml).
  The final cell suspension should comprise roughly 5 × 10^6^ cells per ml.
  IMPORTANT NOTE: TSQ is a diffusible dye. Cells must remain exposed to TSQ during the process of sorting. Removal of dye or significant dilution of the cell sample using PBS can alter staining patterns and reduce the fraction of SC‐β cells labeled by TSQ. Labeling is nearly instantaneous, and there is no need to pre‐incubate dissociated cells with dye before analysis or sorting. In some instances, diluting samples may improve sorting efficiency on flow‐sorting instruments. Ensure appropriate addition of TSQ dye to the cell suspension if dilution is more than five‐fold.
7 Immediately proceed to FACS isolation of SC‐β cells using a sorting setup with protected, sterile conditions in order to maintain a sterile culture environment during sorting. Simultaneously add a 1:100 dilution of 1 mg/ml propidium iodide in order to prevent dead cells from contaminating sorted cells. Collect cells in sorting buffer with a 1:1000 dilution of 10 mM Rho kinase inhibitor stock solution, chilled on ice for the duration of the cytometric experiment.
  TSQ staining requires excitation in the UV/violet spectrum. Using violet rather than UV light sources during sorting can reduce signal. We have also found that PI and TSQ have emission spectra that are difficult to capture in the same gating step. We recommend selecting the TSQ^+^ PI^–^ cell population in two steps: first negatively selecting against PI followed by a TSQ^+^ gate on PI^–^ cells. This is achieved using FSC‐H or another suitable empty channel against each dye alone to avoid patterns caused by spectral overlap in emission ranges.
8 Using a multichannel pipet and sterile trough, plate cell suspension at 5000 cells in 100 µl S3 medium containing 1:1000 dilution of 10 mM stock of Rho kinase inhibitor per well of a 96‐well V‐bottom plate. Spin down plates in a tabletop centrifuge for 5 min at 230 × *g*, room temperature, followed by 24 hr of culture in an incubator at 37°C with 5% CO_2_.
  Size of reaggregates after SC‐β cell enrichment can be manipulated by altering concentration of cells in resuspension. This protocol works well with cluster sizes of 2500 to 15,000 cells per well.
9 24 hr after sorting, replace medium with fresh S3 medium without Rho kinase inhibitor. To re‐feed clusters in plates, spin down each 96‐well V‐bottom plate 5 min at 230 × *g*, room temperature. Aspirate the medium by lowering the pins of an aspirating manifold along the sides of the wells until contact with the edges of the V‐bottom wells is detected.
10 Re‐feed clusters every 48 hr until enriched cells are ready for use.
11 To efficiently recover cells after reaggregation, set a multichannel pipettor to 50 µl higher than the culture volume. Pipet each well up and down, drawing up all medium in each well, depositing reaggregates into an ultra‐low attachment petri dish with PBS. This is best done using eight channels at one time to fit the diameter of a single petri dish. Five thousand cell reaggregated clusters will fit in normal pipet tips. First rinse clean tips with PBS or medium to avoid clusters sticking to the tip wall.

## REAGENTS AND SOLUTIONS

### S3 medium

MCDB 131 basal medium (ThermoFisher, cat. no. 10372019)
8 mM d‐glucose
1.23 g/L NaHCO_3_
2% fatty‐acid‐free bovine serum albumin (Proliant Biologicals, cat. no. 68700)
1:200 insulin‐transferring‐selenium‐ethanolamine (ITS‐X; ThermoFisher, cat. no. 51500056)
2 mM Glutamax (ThermoFisher, cat. no. 35050061)
0.25 mM vitamin C
1% 100× penicillin/streptomycin (ThermoFisher, cat. no. 15140122)
Store up to 1 month at 4°C

From Pagliuca et al. (2014).

### Sorting buffer

Phosphate‐buffered saline (PBS; ThermoFisher, cat. no. 10010002) containing:
2% fetal bovine serum (FBS)
0.5% bovine serum albumin (BSA; Proliant Biologicals, cat. no. 68700)
1:1000 dilution of 10 mM Rho kinase inhibitor stock
125 µM ETDA
Filter sterilize

## COMMENTARY

### Background Information

Purification of stem cell–derived β cells for culture or experimental analysis has been reported recently by several groups, including our own (Nair et al., 2019; Veres et al., 2019). In both of these reports, enrichment of the differentiated endocrine population improves glucose‐stimulated insulin secretion, making this a desirable process for experiments utilizing SC‐β cells from a diverse collection of pluripotent stem cell sources. For this reason, zinc‐based enrichment offers a more rapid and broadly applicable method for enriching SC‐β cells than genetically encoded reporters (Nair et al., 2019). Alternatively, CD49a enrichment is also useful in a number of different backgrounds, although this requires use of antibodies and needs more time and preparation for enrichment. Rapid analysis utilizing flow cytometry–based readouts of SC‐β cells will be easier with TSQ than antibody‐based methods, and is a more adaptable technique to combine with flow cytometry following brief or sequential treatments. In all, TSQ‐based enrichment and imaging of SC‐β cells is a rapid, flow cytometry–based technique for characterization and enrichment of SC‐β cells.

### Critical Parameters and Troubleshooting

Proper labeling of the SC‐β cell population is reproducible using the dye concentrations and methods described here. Unstained controls containing propidium iodide and utilizing empty channels will allow for correct gates to be set during the experiment. Dissociation of SC‐β cells can result in some cell death over time, and we recommend short dissociation times and proper quenching of dissociated cells before resuspension in TSQ‐containing buffer. Inability to utilize UV‐spectrum light to detect the TSQ fluorophore will also reduce the ability to detect and accurately sort SC‐β cells by this method.

### Anticipated Results

In our hands, the TSQ^+^ population closely resembles the total insulin^+^ cell population size resulting from SC‐β cell differentiation protocols. TSQ does not overlap with emission spectra from common fluorophores residing in the green to far red channels, facilitating combination of several different dyes or transgenes with TSQ‐based analysis. Reaggregation of TSQ‐enriched SC‐β cells occurs quickly. Immediately after sorting and plating TSQ‐sorted cells into 96‐well plates, cells form a sheet along the bottom of the well, which quickly forms connections and becomes spherical within 24 to 48 hr. These clusters are durable and easily withstand manipulation with pipetting or repeated wash steps. Clusters of 5000 cells usually generate a single spheroid after reaggregation. Deviation from 5000 cells per cluster can result in multiple clusters in a single well if too many or too few cells are allocated into each well. However, even under these conditions, we do not see significant variability in overall cluster integrity or health of culture. These clusters are stable for several weeks in culture before use.

We generally recover a fraction (<10%) of the starting insulin^+^ population, largely due to inefficiencies from FACS enrichment. This can be overcome by reducing stringency of the flow sorting settings and gates set during the sort, albeit at the cost of overall purity. Efficiency of the sort can also be improved by diluting samples if the abort rate is too high on the sorting instrument. These decisions should be made on a case‐by‐case basis to balance need for large cell numbers with desired purity of the resulting clusters.

### Time Considerations

While TSQ‐based sorting allows for reproducible enrichment of the SC‐β population, cell sorting methods can be time consuming. In our experience, robust differentiations resulting in >30% insulin‐producing cells can yield up to 5 million cells from a starting population of 50 million unenriched cells after 4 hr of sorting time. While preparation of the dissociated cells for sorting is quite rapid, available flow cytometry sorting time and numbers of differentiated cells are limiting factors for maximal cell enrichment and recovery for downstream experiments.
